# 3D Multicolor Super-Resolution Imaging Offers Improved Accuracy in Neuron Tracing

**DOI:** 10.1371/journal.pone.0030826

**Published:** 2012-01-24

**Authors:** Melike Lakadamyali, Hazen Babcock, Mark Bates, Xiaowei Zhuang, Jeff Lichtman

**Affiliations:** 1 Department of Molecular and Cellular Biology, Harvard University, Cambridge, Massachusetts, United States of America; 2 Department of Chemistry and Chemical Biology, Harvard University, Cambridge, Massachusetts, United States of America; 3 Division of Engineering and Applied Sciences, Harvard University, Cambridge, Massachusetts, United States of America; 4 Department of Physics, Harvard University, Cambridge, Massachusetts, United States of America; 5 Howard Hughes Medical Institute, Chevy Chase, Maryland, United States of America; Beijing Normal University, Beijing, China

## Abstract

The connectivity among neurons holds the key to understanding brain function. Mapping neural connectivity in brain circuits requires imaging techniques with high spatial resolution to facilitate neuron tracing and high molecular specificity to mark different cellular and molecular populations. Here, we tested a three-dimensional (3D), multicolor super-resolution imaging method, stochastic optical reconstruction microscopy (STORM), for tracing neural connectivity using cultured hippocampal neurons obtained from wild-type neonatal rat embryos as a model system. Using a membrane specific labeling approach that improves labeling density compared to cytoplasmic labeling, we imaged neural processes at 44 nm 2D and 116 nm 3D resolution as determined by considering both the localization precision of the fluorescent probes and the Nyquist criterion based on label density. Comparison with confocal images showed that, with the currently achieved resolution, we could distinguish and trace substantially more neuronal processes in the super-resolution images. The accuracy of tracing was further improved by using multicolor super-resolution imaging. The resolution obtained here was largely limited by the label density and not by the localization precision of the fluorescent probes. Therefore, higher image resolution, and thus higher tracing accuracy, can in principle be achieved by further improving the label density.

## Introduction

Mapping neural connectivity in the brain is a challenging task [1], [2], [3]. One problem is size: connectivity mapping requires tracing numerous, densely packed axons and dendrites over relatively long distances, while the finest processes can be as small as ∼50 nm. The combination of the high density and the small size of neural processes requires nanometer-scale resolution to disambiguate the closely packed fine processes. A second problem arises from the fact that the function of the synaptic connections is encoded in their molecular profile. Thus, mapping a neural circuit also requires an approach that can provide molecular specificity. Electron microscopy (EM) is commonly used for studying synaptic level details of circuits due to its intrinsically high resolution [4], [5], [6], [7]. However, imaging molecular content at high density with EM is challenging due to the low labeling efficiency of immunogold [8]. Moreover, multicolor coding of different populations of cells, which could help neuron tracing and segmentation, is more difficult with EM.

Fluorescence microscopy, on the other hand, offers high molecular specificity and multicolor imaging capability [9], [10], [11]. To leverage multispectral imaging and distinguish intermixed neural processes of many nerve cells, we previously developed a strategy [12] to tag each neuron with a unique spectral hue. This was accomplished by stochastically expressing within individual neurons multiple spectrally resolvable fluorescent proteins in unique combinations [12]. This “Brainbow” strategy makes long-distance tracing of axons a simpler task due to the consistency of the color that is expressed within each cell [12]. However, due to the small size of neural processes and the diffraction-limited resolution of fluorescence microscopy, it is difficult to unambiguously trace neural connectivity even with the “Brainbow” labeling. Recently, an alternative strategy to neural connectivity mapping via synaptic “Brainbow” labeling has been proposed [13]. Such synaptic “Brainbow” labeling could potentially reduce the resolution requirement and substantially simplify neural connectivity mapping, but its feasibility has yet to be demonstrated experimentally. Moreover, this approach does not provide the ability to trace the morphology of axons and dendrites, which also contain valuable information on how circuits perform computation.

The diffraction limit is no longer an impenetrable barrier to far field fluorescence microscopy. Sub-diffraction-limit fluorescence imaging techniques have been developed [14], [15]. Among these techniques, stochastic optical reconstruction microscopy (STORM) takes advantage of photoswitchable fluorophores to precisely determine the locations of densely distributed molecules [16], [17], [18]. Typically, only a sparse subset of these fluorophores is activated at a time such that the images of the activated fluorophores are non-overlapping and their locations can be determined precisely (with nanometer scale precision). These activated fluorophores are then turned off, another subset activated, and an image is built through iterative cycles of activation and localization. This concept has also been extended to 3D imaging by introducing a cylindrical lens in the optical path such that the ellipticity of the image of each fluorophore is highly dependent on its axial position, whereas the centroid is a measure of the lateral position [19]. Using this approach, resolution of 20 nm in the lateral dimension and 50 nm in the axial dimension has been achieved [16], [19], [20].

When imaging macromolecular structure, the final resolution depends not only on the localization precision but also on the density of label (or the mean separation between neighboring localizations that make up the structure) [21], [22]. While sub-diffraction-limit imaging techniques can intrinsically achieve very high resolution, the labeling density, which depends on the specifics of each sample, can sometimes limit the final resolution. Therefore, it is unknown whether a sufficiently high labeling density could be achieved in neurons to resolve closely packed neural axons and dendrites for connectivity tracing.

Here, we used cultured primary hippocampal neurons as a model system to test whether super-resolution STORM imaging offers improved neural connectivity tracing accuracy as compared to conventional confocal microscopy. The neurons were labeled with fluorescent proteins and subsequently with antibodies against fluorescent proteins. We found that membrane targeted fluorescent protein labeling improved the labeling density, and hence the final image resolution compared to cytoplasmic labeling. With this labeling strategy, we achieved ∼40 nm 2D and ∼110 nm 3D image resolution with STORM, which substantially increased the neurite tracing accuracy over confocal microscopy. Multicolor labeling using the “Brainbow”-like scheme further improved the accuracy of tracing compared to single color labeling. Since the overall resolution achieved here is largely limited by the density of the fluorescence labels, rather than the localization precision of individual fluorophores, we expect that future efforts to increase the label density will lead to higher resolution and more accurate neuron tracing.

## Results

### Methods to Improve Label Density

**Figure 1:**
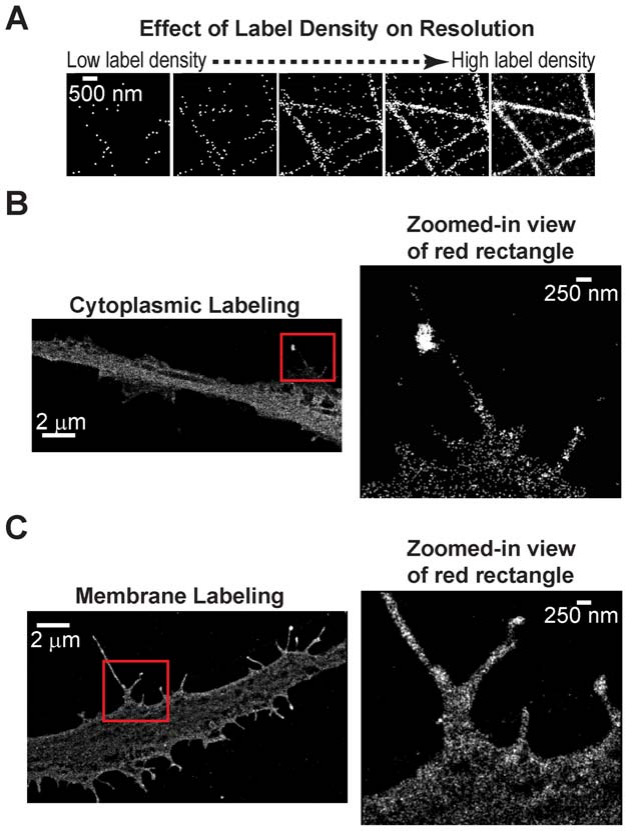
Comparison between cytoplasmic and membrane labeling for neuron imaging. *(A)* STORM images of microtubules demonstrating the effect of label density. In the first panel the localizations from only the first few hundred frames of a STORM movie are included in the reconstructed image to simulate the effect that would be observed in the case of low label density. In the last panel localizations coming from the entire STORM acquisition are included to simulate the effect that would be observed in the case of high label density. The panels in between include progressively increasing number of localizations in the final reconstructed image. It is not possible to reconstruct the actual microtubule structure from the first image due to the low number of localizations, whereas the ability to reconstruct the microtubule structure increases with increasing number of localizations. *(B)* 2D STORM image of a neural process expressing YFP in the cytoplasm. The YFP was immuno-labeled with antibodies conjugated to photoswitchable A405-A647 pair for STORM imaging. The zoomed-in view shows a region with small neural processes. The small volume of these processes results in a low localization density in STORM images. *(C)* 2D STORM image of a neural process expressing mCherry attached to the membrane through a palmitoylation sequence. The mCherry was similarly immuno-labeled with antibodies conjugated to photoswitchable A405-A647 pair. The zoomed-in view shows a region of small neural processes. The membrane targeting resulted in a 3.6-fold improvement in label density.

To trace neurons with high fidelity, the geometrical arrangement of neural processes must be imaged with high resolution. The image resolution of STORM is not only determined by the localization precision of each molecule, but also by the label density. **Figure 1A** shows multiple STORM images of the same structure (microtubules) to demonstrate the effect of label density on image resolution. In the first panel only a few localizations are included in the final image as would be the case if the sample had low label density or if only a small fraction of the label was imaged. The number of localizations included is progressively increased in the subsequent panels. As is evident from these series of images, the fidelity by which the shape of the underlying structure can be reconstructed depends on the number of localizations and therefore the label density. This effect can be quantified by the Nyquist sampling theorem, which states that the obtained resolution is equal to twice the average distance between neighboring labels (see Materials and Methods). Therefore, we tested whether a sufficiently high label density can be achieved when labeling neurons to allow improved resolution by STORM over confocal images of the same sample. Here, we used the strategy of expressing fluorescent proteins in the neurons and then immuno-labeling these fluorescent proteins with photoswitchable dye conjugated antibodies. The antibodies were labeled with a photoswitchable dye pair Alexa 405 (A405) and Alexa 647 (A647) [20]. Antibody labeling can be easily achieved through amine-reactivity of commercially available dyes in a single reaction step ([19], [20], [23]). The subsequent steps of sample preparation involve routine immuno-labeling using standard protocols. An alternative labeling approach for super-resolution imaging would be to directly express photoactivatable fluorescent proteins in neurons. While this alternative approach avoids an additional antibody labeling step, here we chose to use photoswitchable dyes for the following reasons: (i) photoswitchable dyes, such as Alexa 647, are substantially brighter than photoactivatable fluorescent proteins and therefore allow localization of single molecules with higher precision [22]; (ii) the use of immunostaining with photoswitchable dyes allows us to use existing mouse lines expressing conventional non-photoactivatable fluorescent proteins; (iii) multicolor imaging with three or more colors can easily be achieved with photoswitchable dyes [20], [24]; (iv) dyes can be switched off faster than fluorescent proteins without sacrificing photon counts and therefore faster acquisition times can be achieved [22].

To obtain high label density, we first tested several approaches to obtain a high expression level of fluorescent proteins inside hippocampal neurons. Among the two transfection methods used (nucleofection with Amaxa and infection with adenovirus vectors), the viral transfection typically resulted in a higher expression level of the fluorescent protein as noted by fluorescence intensity, which corresponded to a higher label density after immuno-staining and a ∼1.5 fold improvement in the resolution of STORM images. The improvement was assessed by calculating the label density from 30 different areas in several STORM images recorded from three separate sample preparations. Student's t-test showed the difference to be statistically significant with a two-tailed p value of 6*10^−9^. Thus, the viral transfection method was used in the following experiments whenever possible.

We began by imaging neural processes in which the fluorescent proteins were expressed in the cytoplasm and then immuno-stained with a high concentration (0.1 mg/ml) of antibodies. **Figure 1B** shows a 2D STORM image of a cytoplasmically labeled neural process. As shown in the zoomed-in view, thin membrane regions and small neural processes were not well labeled resulting in a low overall label density in these regions (∼680 +/− 200 labels per µm^2^ measured from 18 different regions in multiple STORM images). Based on these results we attempted to improve labeling efficiency by expressing the fluorescent protein on the plasma membrane considering the large surface area to volume ratio of thin neural processes. We targeted the fluorescent proteins to the plasma membrane through a palmitoylation sequence that was appended to the N-terminus of the fluorescent protein via a small linker [25]. **Figure 1C** shows a 2D STORM image of a membrane labeled neural process immuno-stained with a similarly high concentration of antibodies. As is evidenced in the zoomed-in view, small neural processes contained a higher density of localizations (∼2500 +/− 900 labels per µm^2^ measured from 20 different regions in multiple STORM images). This improvement is likely due to the large surface area to volume ratio of thin neural processes. While it is possible that antibody penetration limits the label density in cytoplasmically labeled neurons, this scenario is unlikely since we used membrane permeabilized neurons in both cases. Moreover, the epitopes of the membrane-anchored fluorescent proteins are also likely located on the cytoplasmic side of the membrane. Overall, membrane labeling improved the label density by 3.6 fold compared to the cytoplasmic labeling and therefore the Nyquist resolution by about 1.9 fold in 2D and 1.5 fold in 3D. Therefore, we used the membrane labeling approach for all subsequent experiments unless indicated otherwise.

### Automated Single Color 3D STORM Imaging of Hippocampal Neurons

**Figure 2:**
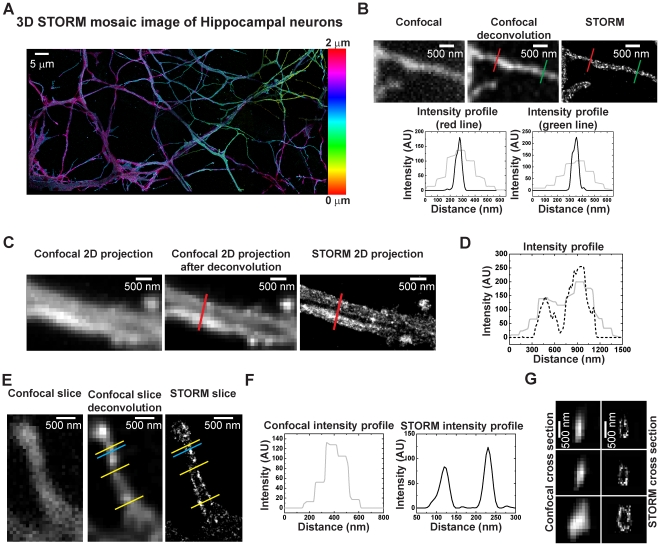
Single color 3D imaging of hippocampal neurons by STORM and confocal. *(A)* Mosaic 3D STORM image of hippocampal neurons. The color indicates z-position information according to the colored scale on the right. This image spans a volume of 147×80×1.4 µm *(B)* A zoomed-in view showing 2D maximum intensity projection of a neural process in confocal (*left*), confocal after deconvolution (*middle*). and STORM *(right)*. The left graph shows the intensity profile in the deconvoluted confocal image (*grey plot*) and the STORM image (*black plot*) across the red line indicated on both images. Similarly, the right graph shows the intensity profile in the deconvoluted confocal image (*grey plot*) and the STORM image (*black plot*) across the green line indicated on both images. The diameter of the neural process at the measured locations is on average 63 nm (FWHM) in STORM and 250 nm (FWHM) in confocal. *(C)* A zoomed-in view showing 2D maximum intensity projection of neural processes imaged by confocal (*left*), confocal after deconvolution (*middle*) and STORM (*right*). Two neural processes in close proximity are resolved in the STORM image but are not as clearly resolved in the confocal image. *(D)* The graphs show the intensity profile plotted across the red line shown in (*C*) for the confocal image after deconvolution (*grey plot*) and the STORM image (*black dotted plot*). Two peaks are visible in the STORM plot indicating the two distinct neural processes in the STORM image. *(E)* xy cross-section of a 100 nm thick slice of a small neural process taken from the midpoint image of a confocal (*left*) and STORM (*right*) stack. The middle panel shows the confocal slice after deconvolution. The membrane boundaries contain more labels and are clearly evident in the STORM slice. *(F)* Intensity profile across the cyan line shown in (*E*) for the confocal image after deconvolution (*grey plot*) and the STORM (*black plot*) image. The two membrane boundaries appear as two well-separated peaks in the STORM plot. *(G)* Vertical cross-section images across the three yellow lines shown in (*E*) for the confocal image after deconvolution and the STORM image. The STORM cross-sections look hollow in the middle, as expected for membrane labeling.

Neural tracing requires tracking processes in 3D and over larger distances than covered by our microscope's field of view. Therefore, we implemented an automated 3D imaging protocol to image neural processes of hippocampal neurons *in vitro* that occupied areas equivalent to many fields of view. In this report, we imaged sample areas of up to ∼20,000 µm^2^. These cultures were relatively flat so that all the processes could be captured by imaging to a depth of ∼1.4 µm (total volume ∼28,000 µm^3^). **Figure 2A** shows a 3D STORM image of the processes of a hippocampal neuron. With our approach the reconstructed volume could be of any size, and was only limited by the time needed to perform the imaging. The mosaic STORM images were aligned by correlating image structures that appeared in overlap regions between adjacent fields of view (see Materials and Methods). With the high labeling density and the low activation powers we could choose to image only a subset of STORM probes in each imaging round, and therefore could reliably correlate the structures in overlap regions for alignment. The error in alignment was estimated to be smaller than the final resolution of our images.

### Resolution Estimate of STORM Images and Comparison with Confocal Images

To estimate the final image resolution, we considered both the localization precision of individual fluorophores and the label density. The latter allowed us to estimate a Nyquist-criterion-based resolution limit that is equal to twice the average distance between neighboring fluorescent labels. Considering that the two contributions (localization precision and Nyquist-criterion-based resolution derived from the label density) represent independent sources of error, we added them in quadrature (according to Equation 2 in Materials and Methods) to calculate the final resolution. The final resolution was estimated to be 44 nm in 2D and ∼110 nm in 3D, and was largely limited by the label density. The 3D resolution was lower than the 2D resolution because the average distance between neighboring labels is larger in 3D than in 2D projections given the same 3D label density. Future efforts to improve label density may bring the resolution closer to what is allowed by the localization precision.

To determine whether STORM provided an improvement over confocal we imaged the same samples with a spinning disk confocal system. We applied deconvolution to the confocal images for the final comparison. Neural processes whose diameter was limited by the diffraction limit in the confocal images (both before and after deconvolution) could be imaged with sub-diffraction resolution in STORM (**Figure 2B**). **Figure 2C** shows zoomed-in comparison of a maximum intensity projection confocal image (before and after deconvolution) and STORM image. **Figure 2D** shows the intensity profile plotted across the red line for both the confocal image after deconvolution and the STORM image. Two neural processes in very close proximity are clearly resolved in the STORM image whereas they blend together in the confocal image. **Figure 2E** shows zoomed-in comparison of a single slice xy-image taken from the mid-plane of a 3D confocal (before and after deconvolution) and STORM stack. In **Figure 2F**, the intensity profile is plotted across the cyan line for the confocal image after deconvolution and the STORM image. The membrane expression of fluorescent proteins is evident in the STORM image as the edges of the neural process are clearly defined whereas the cytoplasm contains fewer labels. This membrane expression pattern cannot be resolved in the confocal image. **Figure 2G** shows a vertical cross-section across the three yellow lines for both the 3D confocal image after deconvolution and the STORM image in **Figure 2E**. The neural processes appear as hollow cylinders in vertical cross-sections of STORM images but not in confocal images. As demonstrated by these examples, the STORM images of neurons showed clear improvement in resolution over confocal both in 2D and in 3D.

### Tracing of Single Color 3D STORM and Confocal Images of Hippocampal Neurons

**Figure 3:**
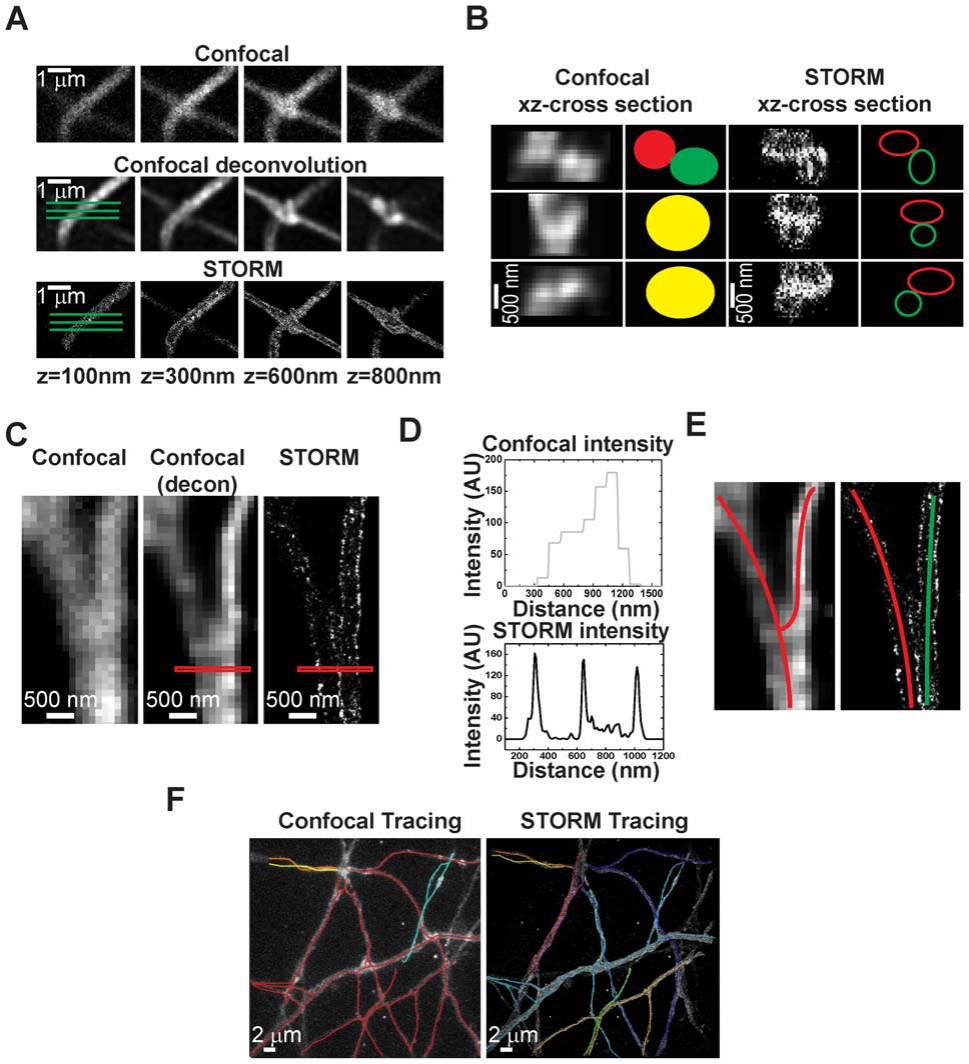
Tracing of hippocampal neurons. *(A)* Z-stack showing two neural processes that cross each other at different heights in confocal (*upper panels*), confocal after deconvolution (*middle panels*), and STORM (*lower panels*). *(B)* The xz cross-sections are plotted across the three green lines in (*A*) for the confocal image after deconvolution (*left*) and the STORM (*right*) image. The xz cross-section of the “top” and “bottom” neural processes cannot be easily discerned in the confocal images at the crossing point (*left*) as they merge together. Thus the two neural processes *(red* and *green circles*) appear to merge into one process (*yellow circles*). On the other hand, the membrane that separates the two neural processes is clear in the STORM cross-sections and a “top” (*red oval*) and “bottom” (*green oval*) neural process can be identified at all locations. *(C)* xy cross-section taken from the midpoint image of a 3D confocal (*left*) and STORM stack (*right*). The middle panel shows the confocal slice after deconvolution. *(D)* The graphs show the intensity profile across the red rectangle shown in (*C*) for the confocal image after deconvolution (*grey plot*) and the STORM (*black plot*) image. Three clearly separable peaks are seen in the STORM plot. The first peak is the membrane edge of the first neuron and the last peak is the membrane edge of the second neuron. The peak in the middle is the membrane boundary that separates the two neurons. *(E)* The difference in tracing results for this region in confocal (*left*) and STORM (*right*). The confocal tracing leads to one parent process splitting into two branches (*red*) whereas the STORM tracing leads to two neural processes (*red and green*) in close proximity. *(F)* Tracing results for an identical region of neurons in confocal (*left*) and STORM (*right*). Distinct processes are assigned different colors.

To assess whether the higher resolution translates into an improvement in tracing accuracy, we followed the same membrane labeled processes in confocal and STORM images. The tracing was done manually. The confocal images were traced first followed by the corresponding STORM images and the same criteria were applied to both images for tracing. The data was in the identical form (3D stacks with 100 nm thick slices) for both confocal and STORM tracing. The processes were mostly followed in x-y using the slice near the middle of the stack and when z information was needed for further clarification (for example at junctions where neural processes cross), additional slices were considered by moving up and down through the stack. One neural process was traced from an end point and followed along its length until it terminated at a second end point. Different colors were assigned to neural processes that were considered distinct. Here, we define two neural processes that never merged as being distinct. Otherwise, we consider the processes to belong to the same neuron and assign them the same color. For example, neural processes that never touched each other in the x-y plane were assigned different colors. Neural processes that touched each other by crossing in the x-y plane (X-shape) were also considered distinct if the 3D stack showed a clear separation in z between a “top” and a “bottom” neural process. **Figure 3A** shows an example in which two neural processes cross each other by forming an X-junction. The images of the two neurons overlap in multiple focal planes in confocal but not in STORM. When the xz-profiles are inspected, even at the exact cross point, the xz-profile of the “top” neuron is clearly separated from that of the “bottom” neuron by a membrane boundary in the STORM image but not in the deconvoluted confocal image (**Figure 3B**). Thus, STORM can separate these neural processes as distinct whereas this separation is not as clear with confocal, leading to two different tracing results (**Figure 3B**). In the case of merging or splitting (Y-shape) (see **Figure 3C**), two neural processes were considered the same if the image showed no clear separation in x-y as well as in z. **Figure 3C** shows an example in which two neural processes form an apparent Y-junction. While the two neural processes appear to merge into one neural process in the confocal image (both before and after deconvolution), the membrane boundary that separates them is clearly visible in the STORM image (**Figure 3C and D**), once again leading to a different tracing result between confocal and STORM (**Figure 3E**).

To quantify the overall improvement in tracing ability, we traced a number of highly intertwined processes in multiple regions. **Figure 3F** shows one example region that was traced using the confocal image as well as the STORM image. In this particular region, 4 neural processes could be identified as distinct in the confocal image whereas the STORM image showed 9 clearly distinct neural processes. We traced three similar regions and found that STORM could distinguish about 2.6 times as many neural processes as confocal could (21 in STORM versus 8 in confocal). However, despite this improvement, some of the neural process still could not be traced for their entire lengths without ambiguities. It should also be noted that the improvement factor over confocal depends on the density of neural processes.

### Multicolor Imaging of Hippocampal Neurons with STORM

**Figure 4:**
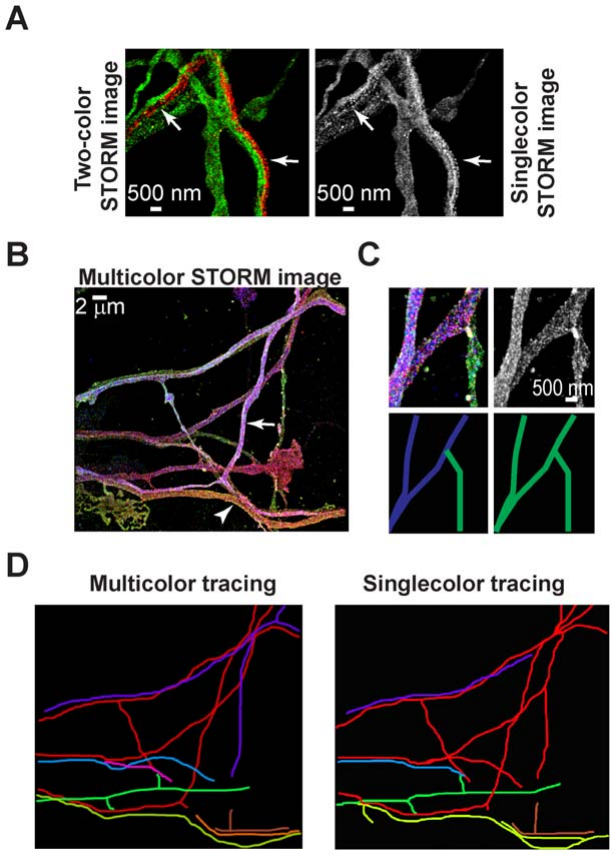
Two-color and multicolor (Brainbow-like) imaging of hippocampal neurons by STORM. *(A)* A zoomed-in field of view of neural processes with (*left*) and without (*right*) the color information. The neurons were separately transfected with YFP and mCherry, mixed and co-cultured. For the STORM imaging, each fluorescent protein was immuno-stained with antibodies conjugated to different dye pairs. Neural processes that are clearly distinct in the two color images (*left, arrows*) are difficult to distinguish in the absence of color (*right, arrows*). *(B)* STORM image of neural processes labeled with a combination of three fluorescent proteins. The neurons were co-transfected with a mixture of the three fluorescent proteins. The co-transfection resulted in co-expression of different amounts of each fluorescent protein inside individual neurons and hence to different color combinations. Each fluorescent protein was immuno-stained with antibodies conjugated to different dye pairs. The arrow and arrowhead point to two neural processes that show different color combinations. *(C)* The STORM image of the same region of neural processes (*upper panels*) is shown in the presence (*left*) and absence (*right*) of color. The tracing results for these two cases are shown in the bottom panels. *(D)* Tracing results with (*left*) and without (*right*) color information for the image shown in (*B*).

To further improve the tracing accuracy, we performed multicolor STORM imaging since neural processes with different colors can be easily identified as being distinct. We first tried two-color imaging with neurons expressing either one of the two colors. To obtain two-color neurons, we transfected the hippocampal neurons separately with either YFP or mCherry prior to plating using nucleofection. We then mixed the YFP and mCherry transfected neurons and co-cultured them (see Materials and Methods). Therefore, each neuron expressed either YFP or mCherry but not a combination of the two. The YFP and mCherry was in turn immunolabeled with A405-A647- and Cy2-A647-conjugated antibodies respectively. The two dye pairs can be distinguished by the wavelength of the light used to activate them, 405 nm and 457 nm light for the A405-A647 and Cy2-A647, respectively [20]. Since we relied on nucleofection, the final resolution of the two-color STORM images were slightly lower than the single color images due to the lower expression levels of the fluorescent proteins (∼70 nm 2D and 178 nm 3D resolution). Nonspecific activation by the imaging laser itself or false activation by the wrong activation laser introduces crosstalk between different color channels in STORM images [26]. False activation by the wrong activation laser is negligible when the A405-A647 and Cy2-A647 pairs are used [20]. Therefore, the main source of crosstalk in this case is the non-specific activation by the imaging laser. Before any crosstalk correction, the crosstalk from mCherry to YFP was 28+/−4% and from YFP to mCherry was 25+/−4%. After subtracting crosstalk due to non-specific activation (see Materials and Methods and [26]), the residual crosstalk from mCherry to YFP was 7+/−2% and from YFP to mCherry was 6+/−2%. As evident in **Figure 4A**, the comparison between two-color and single-color STORM images clearly shows that neural processes in close proximity can be more easily distinguished in the two-color images (arrows).

To increase the number of colors labeling the different neurons, we next tested the compatibility of STORM with a “Brainbow” like labeling scheme. In the “Brainbow” mice, three spectrally different fluorescent proteins are combined in different amounts within each neuron using a Cre-Lox recombination strategy to give rise to a large number of color combinations [12]. To mimic this type of labeling, we co-transfected neurons with a mixture of three fluorescent proteins (YFP, mCherry and tagBFP). In this case tagBFP was cytoplasmic rather than membrane targeted. Since each neuron took up and expressed a different amount of each fluorescent protein, the co-transfection led to several color combinations as observed by epi-fluorescence microscopy (data not shown). We then immuno-stained the neurons with unique primary antibodies against each fluorescent protein followed by corresponding secondary antibodies conjugated to different activator-reporter dye pairs (A405-A647, Cy2-A647 and Cy3-A647) for STORM imaging (see Materials and Methods). The A405-A647, Cy2-A647 and Cy3-A647 pairs can be specifically activated by 405 nm, 457 nm, and 532 nm light, respectively, and the activated A647 fluorophores can then be imaged by the 647 nm light. The non-specific activations by the 647 nm imaging laser (24 +/− 2% per frame) and the false activation of the Cy3-A647 pair by the 457 nm laser (12+/−4%) account for crosstalk between different color channels. The crosstalk contributions were subtracted again as previously described (see Materials and Methods and [26]). After crosstalk subtraction the residual crosstalk among the different color channels was reduced to between 2%-6%. The STORM images of “Brainbow” labeled neurons revealed a range of colors (**Figure 4B**). The ratio of red to green to blue localizations was mostly uniform throughout the same neural process and different among neural processes with different colors. For example, the cyan neuron (arrow) in **Figure 4B** had an average red, green and blue to total localization ratio of 0.29+/−0.02 (red:total), 0.18+/−0.01 (green:total), and 0.52+/−0.03 (blue:total) as determined from measuring the ratios at three locations along its length. The reddish green neuron (arrowhead), on the other hand, had an average ratio of 0.52+/−0.006 (red:total), 0.29+/−0.005 (green:total) and 0.19+/−0.006 (blue:total).

To see if the colors improved the traceability we manually traced the “Brainbow” labeled neurons first in the absence and then in the presence of color information. **Figure 4C** shows an example in which an apparent single neural process in the absence of color was identified as two separate neural processes in the multicolor images. For the region shown in **Figure 4B**, we could trace 8 distinct neurons in the multicolor image as opposed to 6 neurons in the absence of color (**Figure 4D**). Tracing results from three similar regions showed that we could further improve the tracing accuracy by about 40% due to the multicolor labeling (20 versus 14 neural processes traced in three different images). Therefore, given a high density of neural processes, color information can further improve tracing in places where the spatial resolution may not be high enough to resolve nearby neurons.

## Discussion

In this work, we tested the feasibility of using multicolor, 3D STORM to trace neural processes using an *in vitro* model system of cultured hippocampal neurons. We implemented an automated approach to make it easier to image large volumes. We found that 3D STORM allows more accurate tracing of neurons compared to confocal imaging and that membrane labeling is superior to cytoplasmic labeling. We also showed that STORM is compatible with the “Brainbow” labeling, revealing neural processes of cultured neurons with a range of colors at high resolution. Multicolor STORM further surpasses the single color approach in tracing neurons.

We expect that this approach can be used to image and reconstruct neural connectivity in actual brain tissue. This extension will, however, require modifications and improvements of the method. For large volume imaging, serial sections are essential. To reduce tissue loss due to sectioning, brain tissue is often embedded in resins prior to sectioning. It is thus important to develop embedding materials and conditions that preserve the optical properties of fluorophores and/or the antigenicity of the epitopes. Given that the label density is currently the limiting factor in the final resolution obtained here and that even higher resolution would likely be required to trace highly densely packed neurons in the brain, it is particularly important to achieve high label density in brain tissue. Thus, new transgenic lines, viral vectors, and/or labeling methods may need to be developed to provide higher density of fluorescent labels inside individual neurons in brain tissue. Considering our results that membrane labeling gave higher label density than cytoplasmic labeling for the thin processes, it would be desirable to target the fluorescent labels to the plasma membrane.

Another important factor to consider is the imaging speed. The large volume and high resolution required to reconstruct neural circuits demands fast imaging. Recently, we have demonstrated that, a STORM image of a ∼20 µm × 20 µm × 0.5 µm volume can be acquired in 1–10 sec [22]. This speed is comparable or faster than electron microscopy. The scanning speed for scanning electron microscopy (SEM) largely depends on the signal to noise ratio and the resolution, but pixel dwell times on the order of ∼10 µs are typical [5]. Assuming a similar 3D resolution of ∼100 nm, which requires a pixel size of 50 nm, imaging a similar volume by SEM would take 16 sec. Despite the relatively fast STORM imaging speed, imaging a 1 mm^3^ volume of brain tissue would still take on the order of 50–500 days. Thus higher imaging speed is clearly desirable. Potential approaches to improve the imaging speed include developing fluorophores with faster on and off switching rates, developing sensitive cameras capable of faster image acquisition, and developing detection schemes that allow parallel use of multiple cameras.

Overall, we demonstrated with a model system of cultured hippocampal neurons that it is possible to achieve significant improvement in neuron tracing accuracy over confocal by using multicolor, 3D STORM. While the resolution of STORM is not as high as EM, certain advantages of super-resolution fluorescence microscopy (such as multicolor ability and molecular specificity) should prove useful in neural connectivity mapping. Moreover, STORM can, in principle, be combined with EM to leverage the advantages of both techniques.

## Materials and Methods

### Ethics Statement

This study was performed in strict accordance with the recommendations in the Guide for the Care and Use of Laboratory Animals of the National Institutes of Health. The protocol was approved by the Institutional Animal Care and Use Committee (IACUC) of Harvard University (Permit Number: 24-08).

### Preparation of Primary Hippocampal Cultures

Primary hippocampal cultures were prepared from wild-type neonatal (E19) rat embryos (timed pregnant Sprague Dawley rats were obtained from Charles River Laboratories, Wilmington, MA) as described previously [27]. Briefly, around 12 hippocampi were isolated and dissociated using trypsin. The dissociated cells were passed through a 20 µm size filter and plated at a concentration of 1000–5000 cells per 12 mm round poly-D-lysine and laminin coated glass coverslip (BD Biosciences, San Jose, CA). Prior to plating, three round dots of parafilm were positioned onto each coverslip, which acted as feet separating hippocampal neurons from a feeder layer of glial cells. The glia were isolated from rat embryo cortex and grown in culture for a few days in serum-containing media prior to addition of coverslips containing the hippocampal neurons. The glia feeder cells were pre-conditioned with neural growth media (Neurobasal and B 27 from Invitrogen, Carlsbad, CA and a combination of BDNF, CNTF, GDNF and NT3 from Peprotech, Rocky Hill, NJ) a day before plating hippocampal neurons. Neurons were cultured for 7 days for imaging neural morphology. For single color labeling with fluorescent proteins two alternative transfection methods were used. Cells were transfected with fluorescent proteins (mCherry, YFP or TagBFP) either prior to plating using nucleofection with an Amaxa Nucleofector system (Lonza AG, Basel, Switzerland), or after plating and three days prior to fixation using custom made Adenovirus (AV) (Welgen Inc., Worcester, MA). Nucleofection was performed using the Amaxa recommended protocol and 2–3 µg of DNA. The nucleofection system was used for two-color and multicolor labeling. To perform two-color labeling, cells were split into two tubes and each tube was transfected with plasmid DNA encoding one fluorescent protein (YFP or mCherry). After transfection, the cells were mixed and co-cultured. For multicolor “Brainbow” labeling, plasmid DNA encoding either one of mCherry, YFP or TagBFP were combined and cells were co-transfected with this combination. This mixing gave rise to a wide range of combinations of the three fluorescent proteins.

### Preparation of Cells for STORM and Confocal Imaging:

Prior to confocal and STORM imaging cells were fixed using 4% para-formaldehyde and immuno-stained with primary and secondary antibodies as described previously [20]. Secondary antibodies were custom labeled with activator-reporter dye pairs at ratios of about 2 activator dyes and 0.6 reporter dyes per antibody. For YFP, mCherry and Tag-BFP, chicken polyclonal anti-GFP antibody (Aves Labs, Tigard, OR), rabbit polyclonal anti-DsRed antibody (Clontech, Mountain View, CA) and rabbit polyclonal anti-tRFP antibody (Evrogen, Moscow, Russia) were used respectively. Secondary antibodies were purchased from Jackson Immuno Research, West Grove, PA and custom labeled with activator and reporter dyes as described previously [20]. The primary antibodies were tested for their affinity to bind to fluorescent proteins nonspecifically. For example, cells transfected with YFP were stained with antibodies against mCherry or tagBFP. The anti-GFP, anti-tRFP and anti-DsRed antibodies were tested for their binding affinity to the wrong fluorescent protein and this affinity was found to be minimal at concentrations as high as 0.4 mg/ml, 0.04 mg/ml, and 0.02 mg/ml respectively. The affinity was mostly tested by measuring the mean fluorescence signal of the activator dye when the FP was labeled with the right or the wrong antibody. Typically, there was little or no detectable fluorescence when the wrong antibody was used with a given fluorescent protein (mean intensity ∼20–30 times lower than the mean intensity when the right antibody is used). In STORM images, this non-specific binding gave rise to a low label density.

For single color imaging of YFP-transfected cells immuno-staining was performed with polyclonal chicken anti-GFP antibody followed by a secondary antibody against chicken labeled with Alexa Fluor 405 (A405) as the activator and Alexa Fluor 647 (A647) as the reporter [20]. We refer to these antibodies as the A405-A647-labeled antibodies. Likewise, antibodies labeled with Cy2 (or Cy3) as activators and Alexa Fluor 647 (A647) as reporters are referred to as Cy2-A647- or Cy3-A647-labeled antibodies. For two-color imaging, polyclonal rabbit anti-DsRed antibody and a Cy2-A647-labeled secondary antibody against rabbit was used in addition to the chicken anti-GFP/A405-A647-labeled chicken secondary combination described above. For multicolor imaging with three fluorescent proteins, a combination of A405-A647-, Cy2-A647- and Cy3-A647-labeled secondary antibodies were used with their corresponding primary antibodies. In this last case, since two of the primary antibodies were from the same species (rabbit), monovalent Fab fragments (goat-anti-rabbit, Jackson Immuno Research, West Grove, PA) were used to change the species of polyclonal rabbit anti-DsRed from rabbit to goat and thus the immuno-staining was performed sequentially. First the sample was incubated with the rabbit anti-DsRed antibody, followed by a saturating concentration of the monovalent goat Fab fragments, followed by the Cy3-A647 labeled secondary antibody against goat. After extensive washing, the immuno-staining of the Tag-BFP and YFP was carried out together. Following immuno-staining, the cells were placed in a PBS imaging buffer with 100 mM cysteamine (Sigma Aldrich, St. Louis, MO) pH 8.5, 5% glucose (wt/vol) and oxygen scavenging enzymes (0.5 mg ml^−1^ glucose oxidase (Sigma Aldrich, St. Louis, MO), and 40 µg ml^−1^ catalase (Roche Applied Scence, Indianapolis, IN) and mounted for imaging.

### Confocal Imaging

For confocal imaging of hippocampal neurons a Nipkow spinning disk confocal system (CSU series, Yokogawa Electric Corporation, Lake Oswego, OR) was used with a 100X 1.4 NA oil immersion objective (Olympus, Center Valley, PA) and 1.6X zoom lens. To reconstruct a large area, multiple overlapping fields of view were imaged by moving a micrometer stage in x and y. To take a z-stack, the objective was stepped using a nanopositioner (Mad City Labs, Madison, WI) at a 100 nm step size. For YFP imaging, 488 nm light from a water cooled Argon-Krypton laser (Coherent Inc., Santa Clara, CA) was used for illumination. For mCherry imaging, 561 nm light from a solid state laser was used. The fluorescence emission was recorded by an EMCCD Camera (Andor Technology, Belfast, Northern Ireland). Deconvolution of the confocal images was carried out using the 3D Huygens Deconvolution Software from Scientific Volume Imaging and the typical parameters for our microscope.

### STORM Imaging

For STORM imaging of hippocampal neurons, a custom made microscope fitted with a 100X 1.4 NA oil immersion objective (Olympus, Center Valley, PA) was used as described previously [19], [23]. For single color imaging, A405-A647 was imaged using a cycle of 0.2 activation frames (with 405 nm light from a solid state laser, Coherent Inc., Santa Clara, CA) followed by 3 imaging frames (with 647 nm line from an Argon-Krypton laser, Coherent Inc., Santa Clara, CA). The fluorescence emission from A647 was recorded by an EMCCD camera (Andor Technology, Belfast, Northern Ireland) at a frame rate of 60 Hz. The 647 nm light allowed excitation and subsequent deactivation of A647. Excitation with 405 nm light reactivated A647 so that it could be imaged with 647 nm light.

For multicolor imaging, a similar repetitive sequence of activation-imaging (2 frames of activation followed by 3 frames of imaging) was used with multiple activation lasers. 457 nm light from the Argon-Krypton laser was used to activate the Cy2-A647 pair and 532 nm light from a solid state laser (Crystalaser, Reno, NV) was used to activate the Cy3-A647 pair. The localizations were later color coded based on whether the A647 emission was detected after the 405 nm, 457 nm or 532 nm activation light (see Image Analysis). For 3D imaging a cylindrical lens with a 1 m focal length was placed in the emission path [19] to introduce astigmatism.

For the mosaic images that were compared to confocal, STORM imaging was performed following confocal imaging. After confocal imaging, the position of the imaged region was roughly marked using a marker on the glass slide and the sample was moved onto the STORM microscope. The exact field of view was then identified by eye using widefield fluorescence and comparing the view to that of the previous confocal images. The region of interest was then set using a motorized stage to match the confocal imaged region. The cell surface adjacent to the coverslip was used as the starting focal plane. The stage was programmed to record several passes of 50,000-frame partially overlapping movies. A focus lock mechanism (described in [19], [23]) kept the image in focus. Using the cylindrical lens we could detect molecules approximately +/− 400 nm from the focal plane of the objective. In the second and third passes the stage was stepped up by 300 nm using a nanopositioner (Mad City Labs, Madison, WI), and a 50000-frame movie was taken for each field of view. This sequence of three passes was then repeated a second time. For example, for a region of interest consisting of 3 partially overlapping fields of view, 18 movies of 50000 frames each were collected in total (3 images X 3 passes at different focal planes X 2 repeats). The number of frames and the number of passes were chosen to cover the whole z-range of relatively thin neural processes (axons and dendrites) and to exhaustively image all the antibodies labeling the cells. To minimize bleaching of adjacent fields-of-view, a square aperture was placed in front of the illumination path.

### Image Analysis

The raw 2D and 3D, single or multicolor STORM data was analyzed and rendered using custom written software as described previously [16], [19], [20]. Briefly, the image was convolved with a Gaussian kernel to remove high frequency noise and low frequency background. The image was then thresholded and local maxima were identified as peaks. For 2D analysis, the peaks were fit with a 2D Guassian to determine the centroid positions. Sample drift during acquisition was calculated and subtracted by reconstructing STORM images from subsets of frames (typically 500-1000 frames) and correlating these images to a reference frame (typically one that is reconstructed at the initial time segment) [20]. Each localization was rendered as an intensity peak with a Gaussian profile with unit volume, and a width that was scaled to correspond to the theoretical localization uncertainty, based on the number of photons collected for that switching event. For 3D imaging the peaks were fit to an elliptical Gaussian [19]. The x,y position was determined from the centroid as before and the z-position was determined from the x and y widths of the Gaussian function, which were compared to a pre-determined calibration curve [19]. The calibration curve was obtained by imaging single antibody molecules and measuring the x and y width of the images at different z locations by using a nanopositioner to step the stage [19].

For multicolor images, each peak was color coded based on whether the emission was recorded immediately after 405 nm, 457 nm or 532 nm activation cycle. The peaks coming from a frame not belonging to the one right after an activation frame were coded as “non-specific”. In the case of two-color and multicolor imaging, a crosstalk algorithm as described previously was applied to correct for non-specific activations by the imaging laser and false activations by the wrong activation laser [26]. Briefly, we calculated the number of “apparent specific” activations from the frame immediately following the activation pulse and the number of “non-specific” activations from subsequent imaging frames in the imaging cycle. Assuming that the probability of “non-specific” activations is constant across all frames, we could then determine the number of “actual specific” activations by subtracting the “non-specific activation” number from the “apparent specific” activation number. We then used these numbers to statistically subtract crosstalk due to “non-specific” activations in an unbiased way as previously described [26]. Furthermore we also subtracted a small amount of crosstalk between Cy3 and Cy2 (main source of crosstalk due to activation by the wrong activation laser) [26].

The alignment of STORM images taken at multiple stage positions and focal planes was carried out using custom written software. This software first aligned all the STORM data from a single field of view taken at different focal plane positions to make a master data set. Master data sets from adjacent fields of view were then aligned to each other to construct the final STORM image mosaic. Such alignment was necessary as the stage often drifted over time and once moved, the stage did not return to the exact same position.

The initial alignment to construct a master data set was done by rendering 2D images of the STORM data taken at different focal positions. The x, y offsets of these 2D images were then determined by cross-correlation with a reference image, typically the STORM image from the middle focal plane. Next the z offsets between STORM images taken at different focal positions were calculated. Since the z stepping size (300 nm) was smaller than the z detection range (800 nm) at a specific focal position, images taken at different focal positions had overlapped regions between them, allowing the z offset to be calculated by cross-correlation of these overlap regions. Specifically, each STORM image taken at any focal position was rendered as 3D images with 50nm z pixel size. These images were first aligned in x, y as described above. The z offset between the two adjacent 3D images taken at different focal positions was then determined by calculating the cross-correlation of the overlap region in z. The calculated z offsets were then used to align the 3D images in z to generate a master image.

For aligning master images from adjacent fields of view, first the cross-correlation in x and y of the overlap regions was computed and then the cross-correlation in z was computed to determine the z offset. These x, y and z offsets were used to position all the adjacent fields of view to generate the final STORM image mosaic. As the pairwise offsets would often give slightly conflicting optimal positions in data sets with multiple overlapping fields of view, an iterative algorithm was used to minimize the absolute value of the differences in offset. This algorithm worked by recursively moving each field of view towards its local offset difference minima until a convergence criteria was met. The offset data for all the neighbors of a particular field were given equal weight in the minimization. In some cases, the algorithm did not correctly align adjacent fields of view due to the small number of image features in the overlap region between two fields. In these cases the alignment was corrected manually. Alignment of confocal data was performed similarly.

### Calculation of STORM Resolution

The STORM image resolution was calculated by taking into account two factors as described before [22], [28]. First the x,y and z localization precision was calculated by considering clusters of localizations arising from single antibodies bound to the coverslip and finding the center of mass of each cluster. The center of masses of many small clusters were aligned and the x, y and z positions of all the localizations were plotted. The full width half maximum of the resulting Gaussian distribution gives the localization precision in x,y (∼18 nm) and z (∼50 nm).

The second factor considered was the resolution limit due to the finite label density. According to the Nyquist sampling theorem, the maximum resolution obtainable from a given sample is equal to twice the mean distance between neighboring labels in the sample [21]. To calculate the Nyquist resolution limit due to label density in 2D, the 3D data was compressed into 2D. The number of localizations within a small area was calculated for many neural processes in several images recorded from multiple sample preparations. The total number of localizations was then divided by the total area to give the localization density. In principle localization density is not the same as the actual label density since each fluorophore can undergo multiple switching cycles and give rise to multiple localizations. Although multiple localizations from the same fluorophore can substantially increase image quality [24], here we conservatively estimate the Nyquist resolution limit based on the actual label density, which can be obtained from the localization density divided by the average number of localizations that an individual fluorophore gave under our experimental conditions (n = 4). The 2D Nyquist resolution resulting from the label density was then calculated by using the equation:
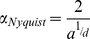
(1)where, *α*_Nyquist_ is the resolution, *a* is the label density and *d* is the dimension (in this case *d* = 2) [21]. The x-y localization precision was then convolved with the Nyquist resolution limited by the label density according to equation (2) to obtain a final 2D image resolution (∼44 nm):
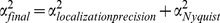
(2)


To calculate 3D resolution from label density, the data was split into a stack of many slices with a thickness of 100 nm. The number of localizations within a small volume was calculated for many neuronal processes in several different STORM images recorded from multiple sample preparations. The total number of localizations was then divided by the total volume and the average number of switching cycles per fluorophore to obtain the label density. The resolution due to label density was calculated using equation (1) with *d* = 3. This number was then convolved with the localization precisions in xy and in z to calculate the xy-resolution (∼105 nm) and z-resolution (∼116 nm) in 3D, respectively.
